# Meta-analysis of randomized controlled trials on vitamin D supplement and cancer incidence and mortality

**DOI:** 10.1042/BSR20190369

**Published:** 2019-11-12

**Authors:** Xinran Zhang, Wenquan Niu

**Affiliations:** Institute of Clinical Medical Sciences, China-Japan Friendship Hospital, Beijing, China

**Keywords:** cancer incidence, cancer mortality, meta-analysis, vitamin D

## Abstract

**Objectives:** We aimed to meta-analyze the results of published randomized controlled trials to test the hypothesis that low vitamin D supplement is associated with an increased risk of cancer incidence and mortality.

**Methods:** Randomized controlled trials that explored the association between vitamin D supplement and cancer incidence or mortality as primary outcomes were identified through searching the PubMed and EMBASE. Literature search and data extraction were performed independently and in duplicate.

**Results:** Ten randomized controlled trials pooled in 81362 participants. The incidence rate of cancer was 9.16% (3716 cases) and 9.29% (3799 cases) in vitamin D intervention group and placebo group, respectively, resulting in a nonsignificant relative risk (RR) (95% confidence interval (95% CI)) of 0.99 (0.94–1.03) (*P*=0.532). The mortality rate of cancer was 2.11% (821 cases) and 2.43% (942 cases) in vitamin D intervention group and placebo group, respectively, resulting in a significant reduction in risk (RR = 0.87, 95% CI: 0.79–0.95, *P*=0.003). There was no observable heterogeneity or publication bias. Subgroup analyses revealed that history of cancer, extra use of vitamin D and calcium supplement were potential sources of heterogeneity.

**Conclusions:** Our findings support a beneficial effect of vitamin D supplement on lowering cancer mortality, especially in subpopulations with no history of cancer, extra use of vitamin D, or calcium supplement.

## Introduction

Cancer is a worldwide public health challenge because of its high incidence and related mortality. Latest report on the global burden of cancer shows an estimated 18.1 million new cancer cases and 9.6 million cancer deaths in 2018 [1]. A targeted cancer control and prevention strategy hence needs to be developed at a global level to reduce the burden on healthcare resources.

Vitamin D supplement has been widely marketed for its claimed anticancer properties [2]. Vitamin D is a fat-soluble vitamin that can be obtained from the diet or made from sunlight exposure [3], and it can regulate cell differentiation and growth through binding to vitamin D receptor in a majority of body cells. Many cell culture and *in vivo* experiments indicate that vitamin D plays a crucial role in preventing cancer development, progression and mortality [4–7]. A growing body of epidemiologic evidence including ‘gold standard’ randomized controlled trials supports a significant association between vitamin D supplement and elevated risk of cancer incidence and mortality [8–16], yet the results are not often reproducible, likely due to insufficient sample sizes, different study designs, or heterogeneous patient characteristics.

To address the current gaps in our knowledge of vitamin D supplement and derive a more reliable estimate, we aimed to meta-analyze the results of published randomized controlled trials to test the hypothesis that low vitamin D supplement is associated with an increased risk of cancer incidence and mortality. Meanwhile, we explored potential sources of between-trial heterogeneity by performing subgroup and meta-regression analyses.

## Methods

This meta-analysis was conducted according to the guidelines from the Preferred Reporting Items for Systematic Reviews and Meta-analyses (PRISMA) statement [17]. The PRISMA checklist is shown in Supplementary Table S1.

### Literature search

A comprehensive literature search, limited to the English language and humans, was manually and independently done by both the authors (Xinran Zhang and Wenquan Niu) through reviewing PubMed (1948–2018) and EMBASE (1974–2018) databases to look for randomized controlled trials. The latest search was undertaken on 17 August 2018. The search terms included (vitamin D OR cholecalciferol OR ergocalciferol OR 25-hydroxyvitamin D OR 1,25-dihydroxyvitamin D OR 1-alpha-hydroxylase OR 25-OH-D OR calcidiol OR calcitriol), AND (cancer OR carcinoma OR tumor OR tumour OR neoplasm) AND (incidence OR mortality) AND words beginning with ‘random’. Cancer incidence or mortality was assigned the primary clinical outcomes. Additional trials were identified in the references of major retrieved articles, including previous relevant systematic reviews and meta-analyses [18–21].

### Inclusion and exclusion criteria

Clinical trials were included if they were published and described as randomized, if they assessed the association of vitamin D supplement with the incidence and/or mortality of all cancer, and if they provided relative risk (RR) or crude data to generate RR. If multiple published reports from the sample clinical trial were available, we selected the publication that most fully covered the intervention period or had the most detailed information.

Ecological studies, cohort studies, case–control studies, reviews, comments or editorials, case reports or series, study protocols, conference abstracts or studies published in languages other than English were excluded.

### Quality assessment

The quality of each eligible randomized controlled trial was assessed using the modified Jadad scoring system [22]. Quality assessment was performed in duplicate and independently by both the authors (Xinran Zhang and Wenquan Niu), and any discrepancies were resolved through discussion.

### Data extraction

From each eligible clinical trial, following data were extracted manually and independently by both the authors (Xinran Zhang and Wenquan Niu) using a standard Excel spreadsheet template (Microsoft, Redmond, WA, U.S.A.): first author’s full name, year of publication, country where trials were conducted, trial name, study characteristics, follow-up duration, 25(OH)D level at both baseline and follow-up, dose of vitamin D or calcium supplement, cancer type, comparison, vitamin D dose, calcium dose, and cancer incidence or mortality in participants with vitamin D or placebo. During data extraction process, any discrepancies were resolved through discussion, and the concordance was 100%.

### Statistical analyses

Summary RR and its 95% confidence interval (95% CI) were calculated to quantify the association of vitamin D supplement with cancer incidence and mortality, separately. Between-trial heterogeneity was assessed by the inconsistency index (*I*^2^) statistic that denotes the percentage of observed variability between trials that is due to heterogeneity rather than chance. The significance cutoff of *I*^2^ statistic was set at 50%, with a higher value denoting a greater degree of heterogeneity [23]. Generally, a fixed-effects model is applied in the absence of between-study heterogeneity, and otherwise the random-effects model. In fact, fixed-effects model only considers sampling error, while random-effects model considers both sample error and between-study diversity [24]. So, random-effects model is more conservative and has a wider CI than fixed-effects model. In this meta-analysis, we adopt the random-effects model for all comparisons irrespective of heterogeneity due to its accommodation to the possibility that underlying effect differs across studies.

Potential sources of between-trial heterogeneity were explored by predefined subgroup analysis (according to gender, history of cancer, extra use of vitamin D, and calcium supplement, respectively) and meta-regression analysis.

The likelihood of publication bias was evaluated by the visual Begg’s funnel plot and filled funnel plot, and by the statistical Egger’s regression asymmetry test with the significance level set below 10%. The Duval and Tweedie nonparametric ‘trim and fill’ method was adopted to account for potential missing studies and derive statistically unbiased estimates.

Above statistical analyses were completed using STATA/SE version 14.1 (StataCorp, College Station, TX, U.S.A.).

## Results

### Eligible trials

**Figure 1:**
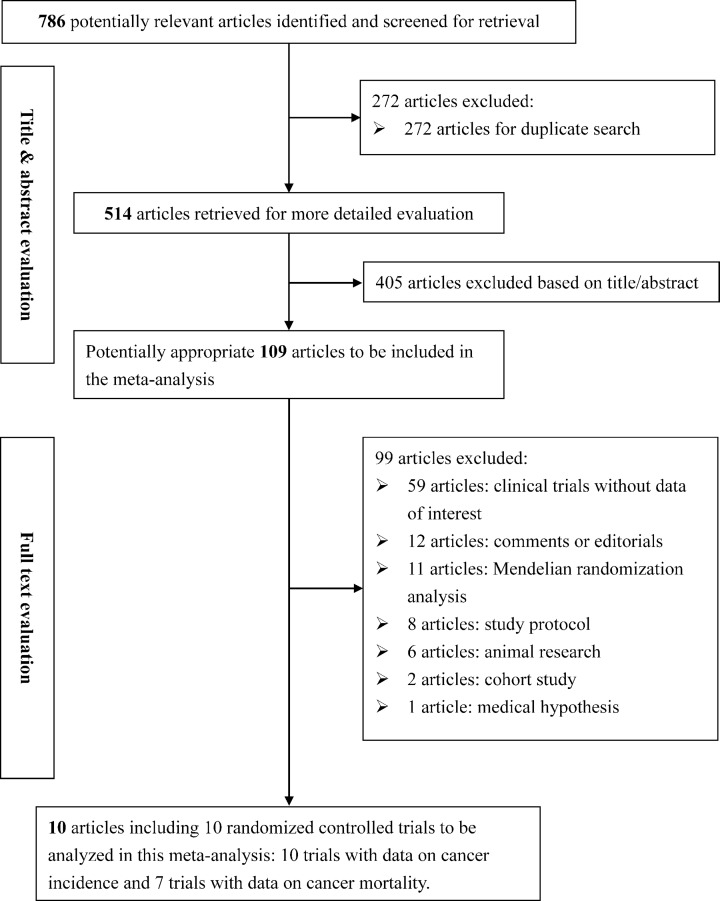
Flow diagram for article selection with specific exclusion reasons

Flow diagram of search strategy and trial exclusion with specific reasons is shown in Figure 1. Using predefined search terms, 786 potentially relevant articles were identified, and only ten articles were eligible for inclusion in this meta-analysis [13–16,25–30], corresponding to ten randomized controlled trials and a total of 81362 study participants. All eligible trials reported data on cancer incidence, and seven (including 77653 study participants) of them reported data on cancer mortality.

### Trial characteristics

Table 1 presents the baseline characteristics of all eligible trials. Trial duration ranged from 48 to 113 months. Three trials involved solely female gender. Quality assessment for each trial is provided in Supplementary Table S2.

**Table 1:** Baseline characteristics of nine randomized controlled trials in this meta-analysis

First author	Year	Country	Cancer	Trial	Population characteristics	25(OH)D level at baseline and follow-up	Eligibility criteria on supplement use	Trial duration (months)	Contrast	Vit D3 dose	Ca dose
Daksha P. Trivedi	2003	U.K.	All cancer	NA	General population aged 65–85 years	Intervention: NA to 74.4 nmol/l at 4 years; control: NA to 53.4 nmol/l at 4 years	Exclude Vit D supplement users	70	Vit D3 vs placebo	100000 IU per 4 months over 5 years	NA
Jean Wactawski-Wende	2006	U.S.A.	Colorectal cancer	NCT00000611	Postmenopausal women aged 50–79 years	NA	Allow for non-protocol supplement of Vit D up to 600IU per day; of Ca up to 1000 mg per day	84	Vit D3 plus Ca vs placebo	400 IU per day for 7 years	1000 mg per day for 7 years
Joan M. Lappe	2007	U.S.A.	All cancer except skin cancer	NCT00352170	Healthy postmenopausal women aged >55 years	Intervention: 71.8–96 nmol/l at 1 year; control: 72.1 to 71.1 nmol/l at 1 year	Not specified	48	Vit D3 plus Ca vs placebo	1100 IU per day	1500 mg per day
Alison Avenell	2012	U.K.	All cancer	RECORD Trial	70 years and older with previous low-trauma fracture	Intervention: 38-62 nmol/l at 1 year; control: 38 to NA nmol/l	Allow for non-protocol supplement of Vit D up to 200IU per day; of Ca up to 500 mg per day	98	Vit D3 (w/t, w/o Ca) vs no Vit D3 (w/t, w/o Ca)	800 IU per day for 24-62 months	1000 mg per day
John A. Baron	2015	U.S.A.	Colorectal adenomas	NA	Age 45–75 years with at least one colorectal adenoma removed within 120 days before enrollment and had no remaining polyps after a complete colonoscopy	NA	Allow for non-protocol supplement of Vit D up to 1000IU per day; of Ca up to 400 mg per day; Excluded patients with 25(OH)D level lower than 12 ng/ml or higher than 90 ng/ml	113	Vit D3 plus Ca vs placebo	1000 IU per day	1200 mg per day
Hans-Christian Pommergaard	2015	Europe, Russia, U.S.A.	Colorectal adenomas	NCT00486512	Patients with one or more sporadic adenomas removed from the colon or rectum within the last 3 months	NA	Exclude Vit D supplement users	72	Calcitriol vs placebo	0.5 μg per day for 3 years	NA
Joan M. Lappe	2017	U.S.A.	All cancer except nonmelanoma skin cancer	NA (NCT01052051)	Postmenopausal women aged ≥55 years	Intervention: 33.0-42.5 ng/ml; control: 32.7–30.9 ng/ml	Allow for non-protocol supplement of Vit D up to 800IU per day; of Ca up to 1500 mg per day	48	Vit D3 plus Ca vs placebo	2000 IU per day	1500 mg per day
Tadashi Akiba	2018	Japan	stage IA-IIIA NSCLC	UMIN	Age: 20–75 years	Intervention: 21-39 ng/mL; control: 22-24 ng/mL	Exclude Vit D supplement users	96	Vit D3 vs placebo	1200 IU per day for 12 months	NA
Robert Scragg	2018	U.S.A.	All cancer except nonmelanoma skin cancer	ViDA study	Age: 50–84 years	Intervention: NA to 25.5 ng/ml; control: NA to 25.2 ng/ml	Exclude Vit D supplement users	57	Vit D3 vs placebo	initial bolus dose of 200000 IU and followed by monthly doses of 100000 IU for a median of 3.3 years (2.5–4.2 years)	NA+A1:N10
JoAnn E. Manson	2018	U.S.A.	All cancer	NCT01169259	General population with men aged 50 years or older and women aged 55 years or older	Intervention: 74–104 nmol/l at 1 year/ control: NA	Allowed for non-protocol supplement of Vit D up to 800IU per day	73	Vit D3 vs placebo	2000 IU per day for 5 years	NA

Abbreviations: Ca, calcium; NA, not available; NSCLC, non-small-cell lung cancer; Vit D, vitamin D; w/t, with; w/o, without.

### Overall analyses

**Figure 2:**
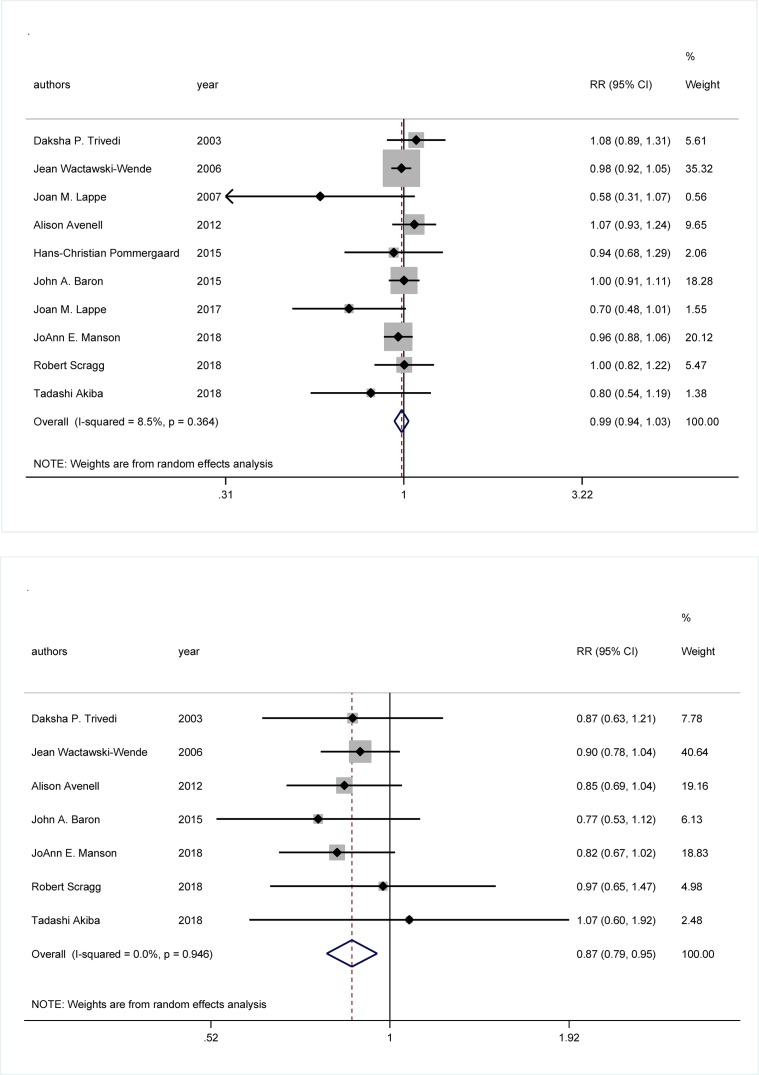
Overall forest plots for both cancer incidence (the upper panel) and cancer mortality (the lower panel) RR is denoted by the center of a solid diamond surrounded by gray box representing the weight of each study, and the length of solid line crossing this diamond denotes its 95% CI. The hollow diamond with a vertical broken line denotes overall risk estimate. The solid vertical line is set at the null value (RR = 1.0).

Shown in Figure 2 are overall forest plots for cancer incidence (ten trials) and cancer mortality (seven trials). The incidence rate of cancer was 9.16% (3716 cases) and 9.29% (3799 cases) in vitamin D intervention group and placebo group, respectively, resulting in a nonsignificant RR (95% CI) of 0.99 (0.94–1.03) (*P*=0.532). There was a statistical low probability of between-trial heterogeneity (*I*^2^ = 8.5%, *P*=0.364) for cancer incidence. The mortality rate of cancer was 2.11% (821 cases) and 2.43% (942 cases) in vitamin D intervention group and placebo group, respectively, resulting in a significant reduction in risk (RR = 0.87, 95% CI: 0.79–0.95, *P*=0.003). Similarly, there was no evidence of heterogeneity (*I*^2^ = 0%, *P*=0.946) for cancer mortality. After excluding the first publication, the RR (95% CI) for cancer incidence and mortality was 0.98 (0.93–1.03) and 0.87 (0.79–0.96), respectively.

### Subgroup analyses

Although there was no statistical heterogeneity in overall analyses, the probability of clinical heterogeneity cannot be excluded. To account for clinical heterogeneity, a panel of subgroup analyses by gender, history of cancer, extra use of vitamin D, and calcium supplement were conducted, respectively (Table 2).

**Table 2:** Subgroup analyses of vitamin D supplement for cancer incidence and mortality

Variables	Subgroups	Studies (*n*)	RR	95% CI	*P*	*I*^2^ (%)	*P*_Heterogeneity_
**Incidence**							
Gender	Both men and women	7	1.00	0.95–1.06	0.957	0.0	0.750
	Only women	3	0.80	0.58–1.11	0.188	65.9	0.053
History of cancer	Without	7	0.99	0.94–1.04	0.667	4.9	0.390
	With	3	0.92	0.76–1.11	0.394	43.5	0.171
Extra use of vitamin D	Without extra use of vitamin D	5	0.98	0.91–1.05	0.964	0.0	0.688
	With extra use of vitamin D	4	0.99	0.92–1.07	0.844	35.1	0.202
Ca supplement	Without	9	0.99	0.95–1.03	0.566	0.0	0.540
	With	1	0.58	0.31–1.07	0.083	NA	NA
**Mortality**							
Gender	Both men and women	6	0.85	0.76–0.96	0.009	0.0	0.923
	Only women	1	0.90	0.78–1.04	0.140	NA	NA
History of cancer	Without	6	0.88	0.80–0.97	0.007	0.0	0.941
	With	1	0.77	0.53–1.12	0.167	NA	NA
Extra use of vitamin D	Without extra use of vitamin D	4	0.87	0.75–1.02	0.090	0.0	0.793
	With extra use of vitamin D	3	0.87	0.78–0.97	0.016	0.0	0.720
Ca supplement	Without Ca supplement	4	0.87	0.75–1.02	0.090	0.0	0.793
	With Ca supplement	3	0.87	0.78–0.97	0.016	0.0	0.720

Abbreviations: Ca, calcium; NA, not available; Vit D, vitamin D.

For cancer incidence, no significance was observed in all predefined subgroups with none or mild heterogeneity. By contract, vitamin D supplement was associated with a significantly reduced risk of cancer mortality in subgroups with both genders (RR = 0.85, 95% CI: 0.76–0.96), with no history of cancer (RR = 0.88, 95% CI: 0.80–0.97), extra use of vitamin D (RR = 0.87, 95% CI: 0.78–0.97), and calcium supplement (RR = 0.87, 95% CI: 0.78–1.02), with nonsignificant heterogeneity (Table 2).

### Meta-regression analyses

**Figure 3:**
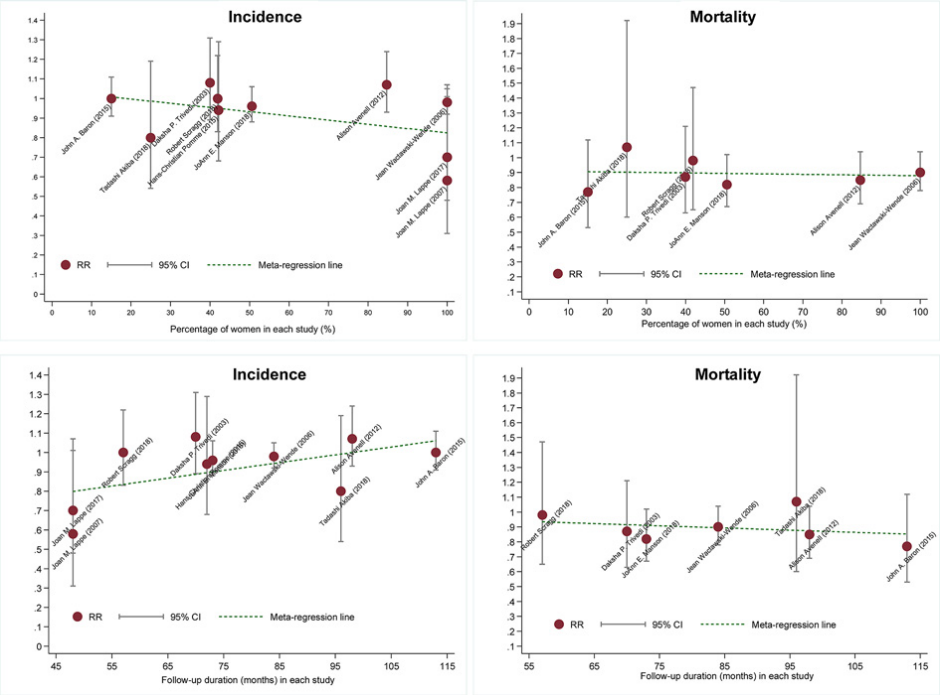
Meta-regression analysis of cancer incidence and cancer mortality according to the percentage of women (the upper panel: incidence left and mortality right) and follow-up duration (the lower panel: incidence left and mortality right) The dark red solid circle represents RR of each individual study, with the black vertical line across the circle representing 95% CI. The green dotted line represents the linear fitted line.

To account for clinical heterogeneity, a meta-regression analysis was conducted by modeling women percentage and follow-up duration of each trial, as shown in Figure 3. The RR for cancer incidence exhibited an obviously decreasing trend with the increasing percentage of women (r = −0.66, *P*=0.0375), yet exhibited an increasing trend with the increase in follow-up duration (r = 0.54, *P*=0.046). The RR for cancer mortality stabilized with the increase in follow-up duration.

### Publication bias

**Figure 4:**
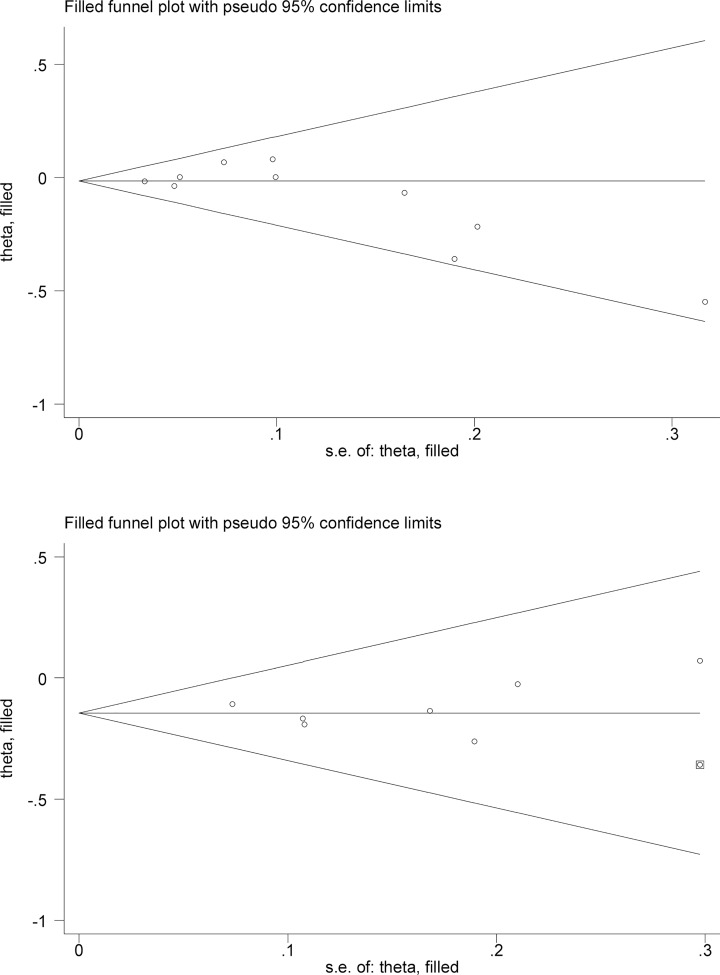
Filled funnel plots for both cancer incidence (the upper panel) and cancer mortality (the lower panel) Hollow circles denote the actual studies included in this meta-analysis, and solid squares denote missing studies required to achieve symmetry of funnel plot.

The probability of publication bias was low for both cancer incidence (*P*_Egger_=0.137) and mortality (*P*_Egger_=0.708) in overall analyses. As reflected by filled funnel plots (Figure 4), visual inspection revealed no missing studies for cancer incidence, and only one missing study for cancer mortality to ensure symmetry. After using the Duval and Tweedie nonparametric ‘trim and fill’ method to account for this missing study, the risk estimates for cancer mortality remained significant (RR = 0.88, 95% CI: 0.79–0.97, *P*=0.010).

## Discussion

Via a comprehensive analysis of ten randomized controlled trials and 81362 participants, our findings support a beneficial effect of vitamin D supplement on lowering cancer-related mortality, rather than cancer incidence. Extending previous studies, we additionally found that cancer history, extra use of vitamin D, and calcium supplement were potential sources of between-trial heterogeneity. To our knowledge, this is thus far the largest meta-analysis of randomized controlled trials that has assessed the association of vitamin D supplement with cancer incidence and mortality.

Low vitamin D status as a risk factor for cancer has been widely evaluated in cohort and case–control studies [31–33], yet data from randomized controlled trials are very limited in the medical literature, and the primary end points in most trials were not cancer incidence or mortality. A previous meta-analysis of four randomized controlled trials by Keum and Giovannucci [18] in 2014 examined the association of vitamin D supplement with cancer incidence and mortality, showing that the benefit of vitamin D supplement may be limited to cancer mortality, in agreement with the major findings identified in this present meta-analysis. The mechanisms underlying the observed significant association between vitamin D supplement and cancer mortality have not yet been fully elucidated. Evidence from animal and *in vitro* studies suggests that vitamin D plays an important role in carcinogenesis, and further affects cancer mortality via several ways, such as reducing cell proliferation, stimulating apoptosis and suppressing cell differentiation of cancer cells. *In vitro* studies indicated that 1,25(OH)_2_D_3_ played an anti-proliferative role in many types of cancer cell based on the induction of cell cycle arrest at the G_1_ phase, and exerted a specific pro-differentiation effect through the induction of differentiation genes [34]. Moreover, vitamin D has many functional indicators, and among them circulating 25(OH)D is the most accurate one as it represents the integrated intake through foods, supplement and exposure to ultraviolet light. Unfortunately, we were unable to analyze the effect of baseline 25(OH)D concentrations, because baseline 25(OH)D concentrations were rarely reported. We agree that further functional explorations are needed to confirm or refute our findings.

Additionally, the limited number of qualified randomized controlled trials in the Keum and Giovannucci study [18] precluded further explorations on heterogeneity, which is a major issue in most quantitative overviews [35]. From the year 2014 onward, several randomized controlled trials on this subject have been published, which prompted us to update the meta-analysis by Keum and Giovannucci [18] to derive a more reliable estimation of the magnitude of the association by integrating ten randomized controlled trials and explore potential sources of between-trial heterogeneity. Besides the similar risk magnitude of incidence and mortality caused by vitamin D, our subgroup analyses revealed that the beneficial effect of vitamin D supplement on cancer mortality was more obvious in subpopulations with no history of cancer, or extra use of vitamin D, or calcium supplement. These subgroup findings are both biologically plausible and supported by previous clinical data. For example, there is evidence for an inverse relation between calcium supplement and cancer risk [36,37], and in this meta-analysis, we found that the effect of vitamin D supplement on cancer mortality was more obvious in participants taking calcium supplement simultaneously, indicating a synergistic interaction or an additive effect between vitamin D and calcium. From a biological viewpoint, high calcium intake can reduce calcitriol concentrations in circulation, which in turn shorten the half-time for serum 25(OH)D—i.e., higher calcitriol concentrations result in greater metabolic consumption and degradation of 25(OH)D, effectively lowering vitamin D status [14]. Nonetheless, given the limited participants in subgroups, our findings should be interpreted with caution and require future validation, especially in randomized controlled trials targeting cancer incidence and mortality as primary end points.

### Limitations

There are several important limitations to the present study that must be acknowledged. First, the majority of randomized controlled trials incorporated in this meta-analysis were conducted in calcium-replete populations, and so the generalization to populations with low calcium intake may be limited. Second, this meta-analysis was restricted to only randomized controlled trials. Although randomized controlled trials can minimize bias and are the gold-standard to infer causality, they may not be reflective of general participants in the clinical practice [38]. Third, as with all meta-analyses, although our analysis revealed a low probability of publication bias, selection bias cannot be completely excluded, as we only searched articles from English journals and published randomized controlled trials.

## Conclusions

Taken together, our findings support a beneficial effect of vitamin D supplement on lowering cancer-related mortality, rather than cancer incidence. More importantly, taking vitamin D supplement will be more efficacious and deserving of recognition as a promising intervention in the subpopulation with no history of cancer, extra use of vitamin D or calcium supplement. For practical reasons, the clinical implication of these findings is that vitamin D supplement can be recommended for cancer patients in current medical practice to offer the potentials for the development of future mortality-reduction strategies.

## Supplementary Material

Supplementary Tables S1-S2

## Abbreviations

*I^2^*: inconsistency index
PRISMA: Preferred Reporting Items for Systematic Reviews and Meta-analyses
RR: relative risk
95% CI: 95% confidence interval
